# Cervicovaginal microbial features predict *Chlamydia trachomatis* spread to the upper genital tract of infected women

**DOI:** 10.1128/iai.00057-25

**Published:** 2025-08-12

**Authors:** Sangmi Jeong, Tammy Tollison, Hayden Brochu, Hsuan Chou, Ian Huntress, Kacy S. Yount, Xiaojing Zheng, Toni Darville, Catherine M. O'Connell, Xinxia Peng

**Affiliations:** 1 Department of Molecular Biomedical Sciences, North Carolina State University 6798, Raleigh, North Carolina, USA; 2 Department of Pediatrics, University of North Carolina at Chapel Hill 549964, Chapel Hill, North Carolina, USA; 3 Bioinformatics Research Center, North Carolina State University 6798, Raleigh, North Carolina, USA; University of California San Diego School of Medicine, La Jolla, California, USA

**Keywords:** cervicovaginal microbiome, *Chlamydia trachomatis*, upper genital tract infection, 16S rRNA sequencing

## Abstract

*Chlamydia trachomatis* (CT) infection can lead to pelvic inflammatory disease, infertility, and other reproductive sequelae when it ascends to the upper genital tract. Factors including chlamydial burden, coinfection with other sexually transmitted bacterial pathogens, and oral contraceptive use influence risk for upper genital tract spread. Cervicovaginal microbiome composition influences CT susceptibility, and we investigated if it contributes to spread by analyzing amplicon sequence variants (ASVs) derived from the V4 region of 16S rRNA genes in vaginal samples collected from women at high risk for CT infection and for whom endometrial infection had been determined. Participants were classified as CT negative (CT−, *n* = 70), CT positive at the cervix (Endo−, *n* = 79), or CT positive at both cervix and endometrium (Endo+, *n* = 68). Although we were unable to identify many significant differences between CT-infected and -uninfected women, differences in abundance of ASVs representing *Lactobacillus iners* and *Lactobacillus crispatus* subspecies but not dominant lactobacilli were detected. Thirteen informative ASVs predicted endometrial chlamydial infection (area under the curve = 0.72), with CT ASV abundance emerging as a key predictor. We also observed a positive correlation between levels of cervically secreted cytokines previously associated with CT ascension and abundance of the informative ASVs. Our findings suggest that vaginal microbial community members may influence chlamydial spread directly by nutrient limitation and/or disrupting endocervical epithelial integrity and indirectly by modulating proinflammatory signaling and/or homeostasis of adaptive immunity. Further investigation of these predictive microbial factors may lead to cervicovaginal microbiome biomarkers useful for identifying women at increased risk for disease.

## INTRODUCTION

*Chlamydia trachomatis* (CT) is the most common, sexually transmitted, bacterial infection globally (1), with young adults at greatest risk for infection. During childbirth, CT can also be transmitted to a newborn through contact with infected cervical tissue and secretions, resulting in infection of mucous membranes of the eye, oropharynx, urogenital tract, and rectum (2). In addition, CT has been extensively studied as a potential co-factor for human papillomavirus (HPV) enhancing cervical cancer risk through cellular transformation, viral load enhancement, and oncogene overexpression (3). When CT ascends from the cervix to the endometrium or uterus, it can lead to upper genital tract infection, increasing risk for pelvic inflammatory disease and reproductive sequelae such as ectopic pregnancy or infertility (2). Despite the availability of diagnostics and effective treatments, the asymptomatic nature of CT infection often leads to undiagnosed cases, contributing to its importance as a global health burden (4).

The cervicovaginal microbiome (CVM) is frequently classified into community state types (CSTs), based on the dominant bacterial species present. Using 16S ribosomal RNA (16s rRNA) gene sequencing, Ravel et al. defined the most common CSTs (I–V) (5). CSTs I, II, III, and V are dominated by specific *Lactobacillus* species with overall lower diversity of other microbiota and are generally associated with healthy reproductive health outcomes (6, 7). However, CST IV comprised heterogeneous, predominantly anaerobic bacterial genera, and *Lactobacillus* species are sparse (5). CST IV is associated with adverse health outcomes such as bacterial vaginosis (BV) (8), pregnancy failure (9), and preterm birth (10). Vaginal dysbiosis, strongly associated with CST IV, has also been linked to increased sexually transmitted infection (STI) vulnerability (7, 8, 11–13). In particular, *in vitro* (14, 15) and *in vivo* (16, 17) studies have associated indole producers within the CVM with CT evasion of interferon (IFN)-mediated depletion of tryptophan, an essential chlamydial nutrient (18, 19). Anaerobes also produce biogenic amines and proinflammatory short-chain fatty acids (SCFAs), promoting symptoms and disease (20, 21). In contrast, *Lactobacillus crispatus*-dominated CST I is considered protective against CT infection (8, 13, 22). Mechanisms contributing to protection include the production of lactic acid that can directly kill CT (23), while D(−) lactic acid produced by some *Lactobacillus* sp. inhibits CT infection by down-regulating host epithelial cell proliferation (6). However, *Lactobacillus iners* synthesizes only L-lactic acid, is less effective at inhibiting pathogen growth, and is weakly associated with protection against CT infection (reviewed by Hand et al. [24]). Other CVM-derived metabolites may also act on their host to stimulate dendritic cell activation and accelerate immunity (25). STI co-pathogens may also act directly, or in conjunction with microbiota, to influence chlamydial infection. We previously observed that women with PID caused by *Neisseria gonorrhoeae* (NG) and CT carried systemic blood transcriptional signatures with greatest activation of cell death pathways and suppression of responses essential for adaptive immunity and CT infection clearance (26). Although these studies have established that CVM composition influences risk for chlamydial cervical infection (8, 13, 22, 27), the extent to which it influences ascending infection has yet to be investigated.

In this cross-sectional study, we sought to investigate the contribution of the CVM to ascending chlamydial infection using high-throughput amplicon sequencing based on 16S rRNA genes by determining if bacterial members, especially those previously associated with susceptibility to cervical infection, can be implicated in chlamydial spread to the upper genital tract. We analyzed the amplicon sequence variants (ASVs) derived from the V4 region of 16S rRNA genes to investigate the CVM of study participants enrolled in T cell Response Against Chlamydia (TRAC), a highly characterized cohort of women at high risk for CT infection and for whom endometrial infection had been determined. When participants were categorized into CSTs based on their CVM composition, CST III (dominated by *Lactobacillus iners*; 30.0%) and CST IV (characterized as diverse; 53.2%) predominated. At the cervical level, ASV analysis revealed no significant overall differences in CVM composition between the CT−, Endo−, and Endo+ groups. Nevertheless, our analysis revealed that certain cervicovaginal microbial features were predictive of ascended chlamydial infection (Endo+). Including CT ASV abundance in the analysis significantly improved prediction accuracy, indicating that CT burden at the cervix is reflective of upper genital tract spread. Furthermore, we detected a positive correlation between microbial features predicting CT ascension and levels of selected cytokines in cervical secretions, indicating that these CVM members may influence infection ascent through their interaction with host immune responses, acting on CT indirectly and directly. This study underscores the potential of developing mucosal biomarkers that could be used to identify women at higher risk of CT upper genital tract infection to improve clinical practice and facilitate future vaccine trials.

## RESULTS

### Classification of participants based on STI clinical diagnostic tests

Cervicovaginal swab samples and endometrial tissue biopsy specimens were collected from 246 women aged 18–35 years in the TRAC cohort (28, 29). All samples were collected before azithromycin and ceftriaxone treatment for CT and NG. Demographics and sexual history of the cohort were previously described (28). Cervical and endometrial infection status of study participants was determined using nucleic acid amplification tests (NAATs) on relevant samples (28). Twenty-six vaginal swab-derived samples were excluded from this study for the following reasons: three had quality concerns identified during DNA extraction, while the infection status of the remaining 23 samples was undetermined because they yielded equivocal results regarding chlamydial detection at the cervix or endometrium during clinical diagnostic testing.

The remaining 220 participants were clinically classified into the following three groups: Endo+, Endo−, and CT− (Table S1). A total of 143 women who tested positively for CT at their cervix (CT+) were subsequently divided into two groups based on confirmed upper genital tract infection. Women testing positively for CT infection at their cervix and endometrium (*n* = 66) were defined as Endo-positive (Endo+) and the remaining CT-infected women who tested positive for CT at their cervix but tested negative for endometrial CT infection (*n* = 77) were grouped as Endo-negative (Endo−). The control group (*n* = 77) comprised women testing negatively for cervical chlamydial infection (CT−). However, among the 77 uninfected women, seven samples from participants that were initially diagnosed as CT− by NAAT clinical testing but subsequently identified as CT+ by the detection of CT reads in 16S analysis were later confirmed to be CT+ by separate quantitation of CT burden in cervical and/or endometrial samples by qPCR (30). Of these seven CT+ participants, two were confirmed Endo+, two were Endo−, and the endometrial infection status for the remaining three could not be determined. Consequently, these seven participants were included in 16S microbiome analyses comparing CT+ to uninfected (CT+, *n* = 150; CT−, *n* = 70), but the three participants with undetermined endometrial infection status were excluded from analyses of features associated with chlamydial upper genital tract infection (Endo+, *n* = 68; Endo−, *n* = 79). Clinical diagnostic testing at enrollment additionally determined cervical infection with the bacterial STI pathogens NG and/or *Mycoplasma genitalium* (MG) in all participants (Table S1) (28).

### Characteristics of cervicovaginal microbiota in the TRAC cohort

**Fig 1:**
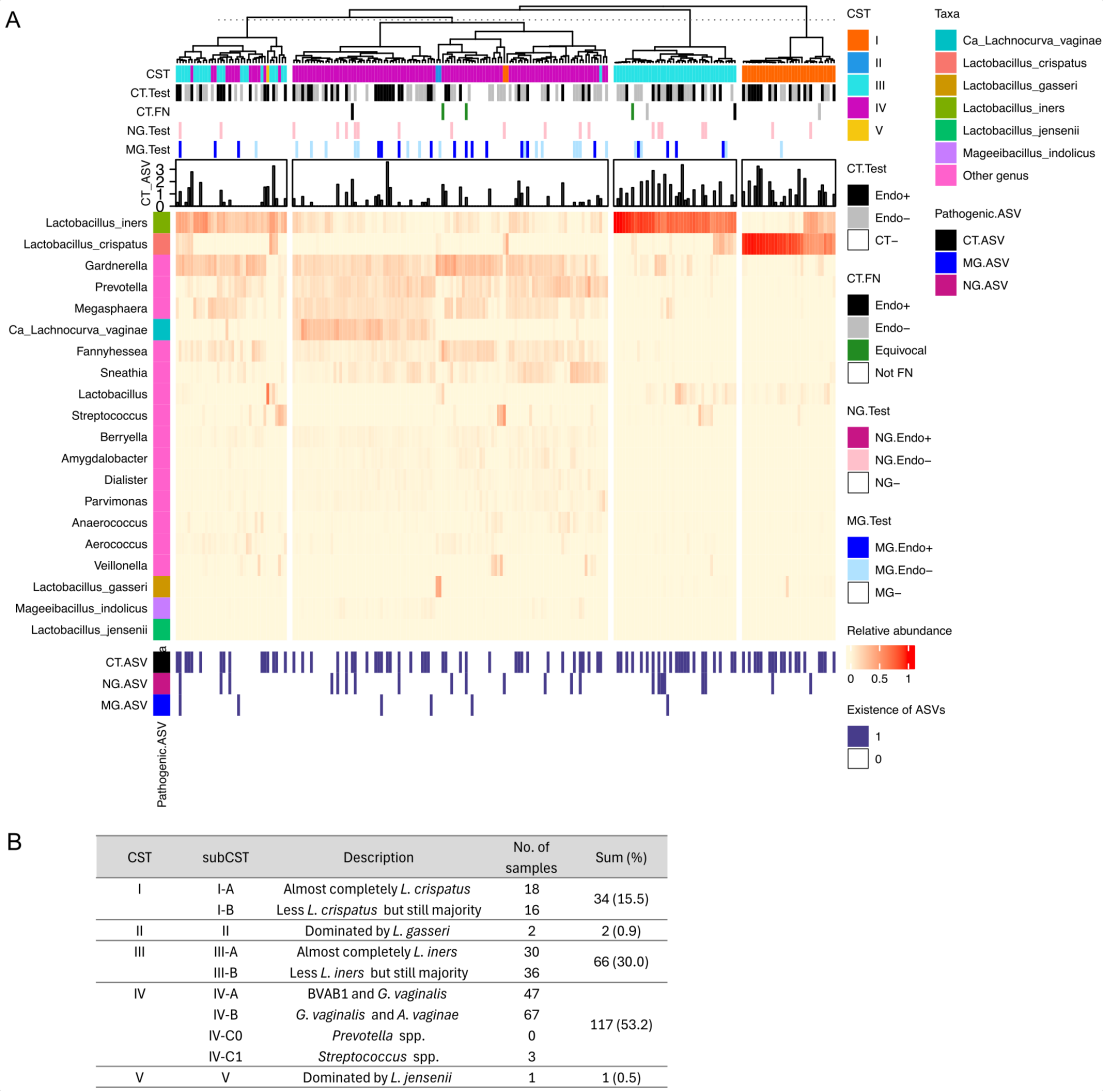
Profiles of community state types from the TRAC cohort associated with chlamydial infection. (**A**) The taxonomic composition of 220 women, incorporating CSTs and pathogen infection status, was presented on the heat map. The testing results for pathogenic infection status at enrollment (CT.Test, NG.Test, MG.Test) were provided as bar graphs on the top of the heatmap. The reassigned results of seven false negatives for CT infection are labeled as CT.FN. The term “equivocal” indicates that the reassignment of these cases as either Endo+ or Endo− was unclear, despite them being confirmed as CT+. CT ASV read counts were converted into logarithmic scale (CT_ASV; log10(read count + 1)). The observations of three pathogenic ASV sequences were illustrated with “existence of ASVs” at the bottom of the heatmap (CT.ASV, NG.ASV, MG.ASV). (**B**) The table shows the definitions of CSTs and the number of women belonging to each CST.

The CVM of TRAC participants were profiled using 16S rRNA gene V4 region sequencing and ASV-based analysis. ASVs identifying common STI bacterial pathogens were detected in cervicovaginal 16S rRNA including CT, NG, and MG (Fig. 1A). CT ASV vaginal abundance among participants testing positively for CT infection was positively correlated with CT cervical burden (Spearman correlation coefficient 0.575) (30) previously assayed by qPCR (28). CT ASVss were detected in 90 of the 150 CT-infected women (60%) (Fig. 1A; Table S1). Of 20 NG-infected women identified by clinical diagnostic testing, NG ASV sequences were detected in 17 of 20 (85%), while NG ASV sequences were detected in 5 out of 200 women testing negatively (2.5%). Assaying via qPCR indicated these discrepancies most likely were a consequence of low burden and/or differential site sampling (30). In contrast, MG-identifying ASV sequences were detected in only 6 of the 38 participants clinically diagnosed with MG (15.8%), an observation explained by the overall low burden of MG detected in infected TRAC participants (31). MG abundance in cervical specimens, previously determined by qPCR (31), was significantly associated with the detection of MG ASV in sample libraries (Mann-Whitney, *P* = 0.0206). Overall, these results support the high quality of this 16S rRNA-based sequencing data set.

Based on ASV relative abundance from 217 women, the most prevalent genera in these women were *Lactobacillus, Gardnerella, Prevotella*, and *Megasphaera* (Fig. 1A; Fig. S1A). The three most abundant ASVs observed overall were *Lactobacillus iners, Lactobacillus crispatus,* and *Gardnerella* spp. (Fig. S1B). Within each group (CT−, Endo−, and Endo+), individuals exhibited a spectrum of *Lactobacillus* abundance, from *Lactobacillus*-predominant compositions to *Lactobacillus*-sparse (Fig. 1A; Fig. S1A and B). Alpha diversity indices were assessed at the ASV level to investigate whether diversities of cervicovaginal microbial communities differed among the Endo+, Endo−, and CT− groups. Neither Shannon nor Inverse Simpson indices were significantly different among three groups (Fig. S1C; Wilcoxon rank-sum test *P*-values >0.05).

### CSTs represented within the TRAC cohort

The cervicovaginal microbial composition of TRAC participants was further evaluated by categorizing each sample according to CST, taxonomic profiles that represent discrete vaginal microbial communities (Fig. 1) (5, 32). The TRAC cohort exhibits a high prevalence of CT infection (68%, 150 of 220 participants) with CST IV (characterized as diverse) as the predominant CST (53.2%, 117 of 220, Fig. 1). Among those assigned to CST IV, 47 participants were classified as CST IV-A (a high to moderate relative abundance of bacterial vaginosis-assciated bacterium 1 (BVAB1) and *Gardnerella vaginalis*), and 67 were classified as CST IV-B (a high to moderate relative abundance of *G. vaginalis* and *Atopobium vaginae*). Almost all the remaining participants were assigned as CST I (dominated by *L. crispatus;* 15.5%, 34 of 220) or CST III (dominated by *L. iners*; 30.0%, 66 of 220) (Fig. 1). Two participants were assigned to CST II (dominated by *Lactobacillus gasseri*), and one participant was assigned to CST V (dominated by *Lactobacillus jensenii*) (Fig. 1). The seven participants who tested false CT negative were assigned to CST I, III, and IV (Table S1).

To assess a potential relationship between CST and epidemiological information in the TRAC cohort, we first evaluated the association between CST and cervical CT infection status for 220 women (Tables S1 and S2). However, CT infection status and CST were not significantly associated (Table S2; Fisher’s exact test *P*-value >0.05). Association between CST and demographics and physiologic information was also assessed (Tables S1 and S2). CST was significantly associated with Nugent score or race (Table S2; Fisher’s exact test *P*-value <0.05). Of the 117 women in CST IV, 106 (90.6%) were classified as having BV based on the Nugent score, compared to lower proportions of BV in the other CSTs. CST IV was more common among Black and multiracial participants, whereas CST I was more common among Caucasian participants, consistent with previous studies (5, 12, 33–36).

### Significant differences in abundance were observed between the CT+ and CT− groups for *Lactobacillus* and *Prevotella* species

**Fig 2:**
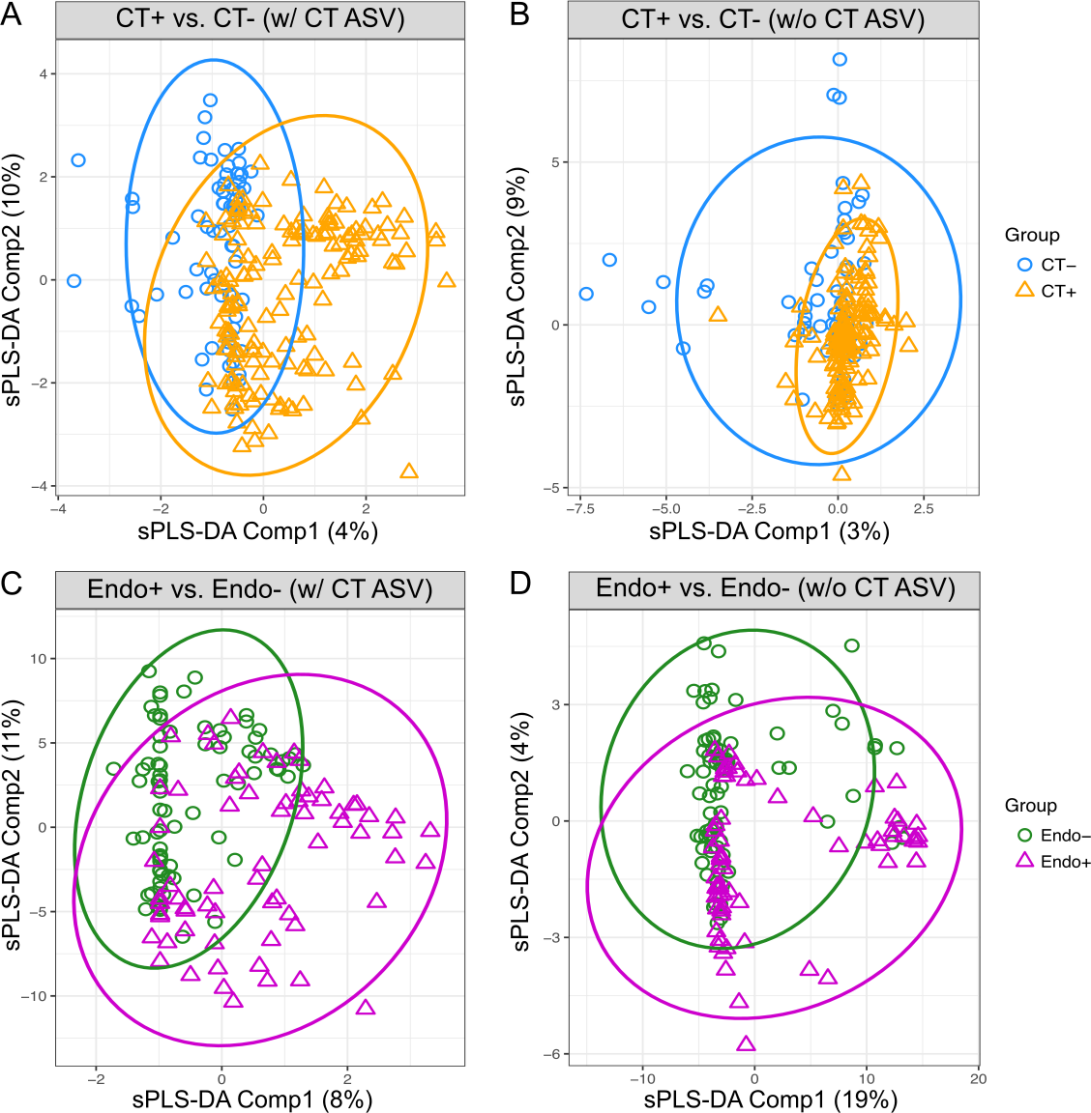
Discrimination of chlamydial infection status at the cervix or endometrium in the TRAC cohort using sPLS-DA. Discrimination of chlamydial infection status at the cervix (**A and B**) and endometrium (**C and D**) was visualized using the first two components of sPLS-DA. The discriminations were performed using ASVs including CT ASV (**A and C**) or excluding CT ASV (**B and D**). Each component’s contribution to the discrimination was indicated at their axis. The performance was evaluated using the mean AUC values. (**A**) The first two sPLS-DA components discriminate CT+ (*n* = 150) from CT− (*n* = 70) women based on ASVs with CT ASV (AUC mean = 0.76, AUC SD = 0.02). (**B**) The discrimination of CT+ from CT− women is shown based on the first two sPLS-DA components, without CT ASV (AUC mean = 0.48, AUC SD = 0.03). (**C**) The first two sPLS-DA components discriminate Endo+ (*n* = 68) from Endo− (*n* = 79) women (AUC mean = 0.74, AUC SD = 0.01). (**D**) The discrimination of Endo+ from Endo− women is shown based on the first two sPLS-DA components, without CT ASV (AUC mean = 0.55, AUC SD = 0.03).

Since it has been reported that cervicovaginal microbial profiles differ between CT− and CT + individuals (37–41), we investigated CVM compositions of CT+ and CT− TRAC participants. We used sparse partial least squares discriminant analysis (sPLS-DA) (42) because this method is effective for supervised classification and feature selection, aiming to identify the best discriminating features between groups while handling high-dimensional data. The statistical discrimination performance for classifying CT− and CT+ women, using the first two sPLS-DA components, was estimated by a mean area under the curve (AUC) of 0.76 (Fig. 2A), indicating that overall CVM profiles in the presence of CT ASV tended to differ between CT+ and CT− women. However, when CT ASV was excluded from the discrimination analysis, performance dropped to a mean AUC of 0.48 (Fig. 2B).

We also performed sPLS-DA within CSTs to examine if differences in CVM composition (excluding CT ASV) could be detected between CT+ and CT− individuals. In CST I (dominated by *L. crispatus*), CT− women appeared to cluster in a manner visually distinct from CT+ women but discrimination performance was statistically inconsistent, with a mean AUC of 0.54, likely due to the small number of samples assigned to CST I (*n* = 34) (Fig. S2A). Within CST III (dominated by *L. iners*), cervical CT infection status was moderately discriminated (AUC mean = 0.64, AUC SD = 0.05, *n* = 66) (Fig. S2B) selecting seven low-abundant *L. crispatus* ASVs for the first sPLS-DA component (Table S3). Within CST IV-assigned participants, the discrimination between CT+ and CT− groups was weak (AUC mean = 0.47, AUC SD = 0.04, *n* = 117) (Fig. S2C).

To identify cervicovaginal microbiome features that are differentially abundant between CT− and CT+ groups, we used Microbiome Multivariate Association with Linear Models (2) (MaAsLin2) (43), which produces a list of significant microbial features with false discovery rate correction. We identified significantly differentially abundant ASVs between CT+ and CT− participants using two approaches: (i) including all women including coinfections (*n* = 220) and including only women not coinfected with NG and/or MG (*n* = 167) (Table S4). A total of 26 ASVs differed in abundance between CT+ (*n* = 70) and CT− (*n* = 150) groups, including ASVs assigned to *Fannyhessea vaginae* and multiple *Prevotella* spp., microorganisms previously associated with enhanced sensitivity to STI and BV (7, 8, 11–13) (Table S4, test results = all women and shared, unadjusted *P*-value <0.05). CT (ASV sequence # ZOtu76) and NG (ASV sequence # ZOtu95) were more abundant in CT+ women, with Benjamini-Hochberg (BH)-adjusted *P*-values <0.0001 and unadjusted *P*-values <0.05, respectively. While a *Criibacterium bergeronii* ASV and two *Stomatobaculum_sp002892395* ASVs (ASV sequence #s ZOtu200, ZOtu1814, and ZOtu1481) were significantly more abundant in CT+ women, *Atopobacter sp003664595*, *Dialister micraerophilus,* and *Lancefieldella parvula* (ASV sequence #’s ZOtu160, ZOtu105, and ZOtu369) were significantly less abundant in CT+ women (Table S4, test results = all women and shared, unadjusted *P*-value <0.05). Three of the 26 ASVs, including NG and *Prevotella* spp. (ASV sequence #s ZOtu95, ZOtu304, and ZOtu1197), were also identified as informative ASVs discriminating cervical CT infection status by sPLS-DA (Table S3 and Table S4).

We also identified 34 ASVs that were significantly differentially abundant between CT+ (*n* = 61) and CT− (*n* = 106) groups, after excluding women coinfected with NG and/or MG (Table S4, test results = non-coinfected only and shared, unadjusted *P*-value <0.05). Among these, 11 ASVs, including three *Prevotella* ASVs (ASV sequence #s ZOtu1197, ZOtu829, and ZOtu304), were already identified as differentially abundant in all women, including those with coinfections (Table S4, test results = shared, unadjusted *P*-value <0.05). In these women without coinfections, *Lawsonella clevelandensis* and *Limosilactobacillus coleohominis* ASVs (ASV sequence #s ZOtu124 and ZOtu61) were significantly more abundant in CT+ women. One *Bulleidia extructa* ASV (ASV sequence # ZOtu88), three *Gemella asaccharolytica* ASVs (ASV sequence #s ZOtu1575, ZOtu413, and ZOtu588), five *Prevotella* spp., and *Candidatus Mycoplasma girerdii* (ASV sequence # ZOtu45) were significantly more abundant in CT− women.

Although no significant differences were detected in the abundance of dominant *Lactobacillus* spp. ASVs (ASV sequence #s ZOtu1, ZOtu2, ZOtu11, and ZOtu32) between CT+ and CT− participants in the overall cohort, differences were observed in 18 uncommon ASVs assigned to *L. iners*, *L. crispatus*, *L. jensenii*, and *L. gasseri* between CT + and CT− either in the overall cohort (*n* = 220) or in the non-coinfected subgroup (*n* = 167) (Table S4; Fig. S3). To investigate if these low-abundance ASVs were sequencing artifacts, we conducted multiple sequence alignments to identify the position of mismatches between these low-abundant *Lactobacillus* ASV sequences and their corresponding reference sequence, reasoning that errors generated by merging of paired-end reads would be disproportionately concentrated in the middle of sequences. The 18 ASV sequences were aligned with the corresponding ASV sequences of their most abundant *Lactobacillus* sp. ASVs listed in Table S4, as well as with 16S reference sequences of *Lactobacillus* sp. from the Ribosomal Database Project (RDP) 16S No18 reference database and *Lactobacillus mulieris* from NCBI RefSeq (NR_180521.1) (44) (Fig. S4). Since the V4 region sequences of *L. mulieris* and *L. jensenii* are identical, it is challenging to distinguish between these two species based on taxonomy assignments. The most abundant *Lactobacillus* ASVs (ASV sequence #s ZOtu1, ZOtu2, ZOtu11, and ZOtu32) exactly matched their corresponding V4 region reference sequences (Fig. S4). In contrast, the 18 low-abundant *Lactobacillus* ASV sequences contained mismatches at 20 of 253 nucleotides in the V4 region. Intriguingly, these nucleotide variants frequently occurred at the same 20 positions along the *Lactobacillus* V4 region rather than being concentrated in the middle of ASV sequences (Fig. S4). These results suggested that the observed sequence variants in these low-abundant *Lactobacillus* ASVs reflected biological variation in *Lactobacillus* subspecies or strains that composed the CVM of TRAC participants.

We investigated whether CVM features could predict CT infection status within this high STI risk cohort, the majority of whom had BV at enrollment (28) (Table S1). We performed a random forest-based analysis using ASV abundance while excluding the CT ASV. In brief, we randomly partitioned the samples from CT+ and CT− groups into a training data set and a separate test data set. We built a predictive model using the training data set and evaluated its performance by predicting the CT infection status using the independent test data set (see Materials and Methods for details). The prediction performance was assessed using the average receiver operating characteristic (ROC) curves across 100 replicates. The corresponding average AUC was found to be 0.6 for the prediction using all ASVs excluding the CT ASV (Fig. S5). Eight ASVs contributed to the prediction of absent CT infection (Table S5). NG ASV (ASV sequence # ZOtu95), more abundant in CT+ women (Table S5; Fig. S6), was the strongest contributor to the prediction. NG is not considered a member of the CVM, thus its contribution to this prediction likely derives from the frequency of coinfection with CT in women reporting high-risk behaviors for STI acquisition. Two *Stomatobaculum sp002892395-*assigned ASVs (ASV sequence #s ZOtu1814 and ZOtu1481) and one *Prevotella* spp. ASV (ASV sequence # ZOtu829), which showed differential abundance between CT+ and CT− women (Table S4), were found to be predictors of absent CT infection (Table S5; Fig. S6). However, the ASVs of *S. sp002892395* (ASV sequence #s ZOtu709, ZOtu1481, and ZOtu1814) have low confidence genus-level assignments, indicating that these strains, currently classified as members of the class *Clostridia*, require further investigation to resolve their taxonomic classification.

### Cervicovaginal microbial features are predictive of susceptibility to ascended chlamydial infection

Given the severe sequelae that can arise after CT ascends to the endometrium and fallopian tubes and our current inability to detect its presence in the upper genital tract non-invasively, we investigated if CVM profiles of CT+ women reflected coincident endometrial infection. We first examined the overall differences in CVM compositions between Endo− and Endo+ patients using sPLS-DA as before (see also Materials and Methods). Endo+ and Endo− women showed distinguishable separations by the first two sPLS-DA components including CT ASV (Fig. 2C; AUC mean = 0.74, AUC SD = 0.01). The CT ASV, two *Lactobacillus* ASVs, and *Actinotignum schaalii* (ASV sequence #s ZOtu76, ZOtu915, ZOtu914, and ZOtu281) contributed the most to the first component, while more than 60 ASVs were identified as important features for the second component discriminating Endo+ and Endo− women (Table S6). Despite the high number of important features identified, in the absence of CT ASV, discrimination between Endo+ and Endo− weakened with an AUC of 0.55 (Fig. 2D).

We also examined the CVM profiles, including CT ASV, within CST I, CST III, and CST IV types separately for CT endometrial infection status using sPLS-DA. Endo− women were visually separable from Endo+ women within CST I, with the first sPLS-DA component including eight *Lactobacillus* ASVs (Table S6), but the statistical performance was low, with a mean AUC of 0.60 (Fig. S2D), again likely due to the small number of women within CST I (*n* = 25). The discrimination of endometrial infection status within CST III was also weak (AUC mean = 0.47, AUC SD = 0.05, *n* = 47) (Fig. S2E). However, endometrial infection status within CST IV (characterized as diverse) can be discriminated (AUC mean = 0.74, AUC SD = 0.03, *n* = 74) (Fig. S2F) with the CT ASV solely contributing to the first sPLS-DA component (Table S6).

**Fig 3:**
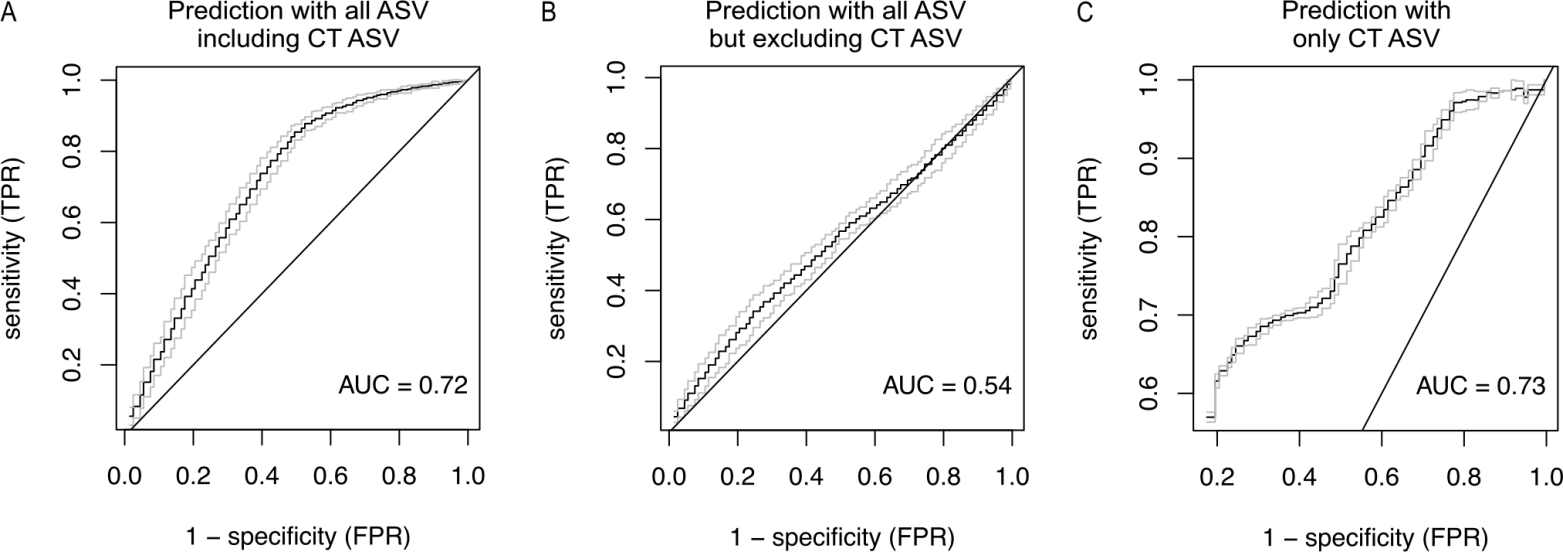
ROC curves and corresponding AUCs from random forest prediction for susceptibility to ascending chlamydial infection. Prediction accuracies were evaluated by averaging AUCs over 100 replicates for each scenario. (**A**) The prediction performance for the susceptibility to CT ascension using all ASVs, including CT ASV, resulted in an AUC of 0.72. (**B**) The prediction performance for excluding CT ASV resulted in an AUC of 0.54. (**C**) The susceptibility of ascending CT infection, predicted using only CT ASV, led to an AUC of 0.73.

To identify CVM features that would be most informative in predicting CT ascension, we performed random forest-based analysis using ASV abundances as features to predict individual patients in Endo+ (*n* = 68) or Endo− (*n* = 79) groups. Since CT is not only a key factor of ascended infection but potentially modulates the cervical and endometrial environment for other microbes, we assessed the predictive performance of the model in three scenarios: (i) prediction with all ASVs including CT ASV (# ZOtu76) (Fig. 3A), (ii) prediction with all ASVs but excluding CT ASV (Fig. 3B), and (iii) prediction with only CT ASV (Fig. 3C). Average AUCs were 0.72 for the model with all ASVs and 0.54 for the model that excludes the CT ASV (Fig. 3A and B), indicating the importance of CT ASV abundance for prediction. When the model was trained by the CT ASV alone, it resulted in an average AUC of 0.73 (Fig. 3C).

In the prediction with all ASVs including CT ASV, CT and 12 additional ASVs were identified as informative features using selection frequency based on mean decrease in accuracy (Table 1; feature selection frequency 80 out of 100 replicates). These 12 ASVs were also selected as informative features in the prediction using all ASVs but excluding CT ASV (Table 1; feature selection frequency ≥ 80 out of 100 replicates). Notably, there was no overlap between these features and those selected as predictors of absent CT cervical infection (Table S5). Among the identified 13 ASVs, 4 were identified with higher abundance in Endo+ women (ASV sequence #s ZOtu76, ZOtu233, ZOtu1808, and ZOtu2665) (Table 1; Fig. S7). These four highly abundant ASVs in Endo+ women were also identified as important features discriminating Endo+ and Endo− patients in the above sPLS-DA (Table 1 and Table S6). Two ASVs assigned to *Sutterella sp900762445* (ASV sequence #s ZOtu110 and ZOtu184) exhibited lower abundance in Endo+ women (Table 1; Fig. S7). *Actinotignum schaalii* ASV (ASV sequence # Zotu281), *Actinobaculum massiliense* (ASV sequence # ZOtu567), *Haemophilus haemolyticus* ASV (ASV sequence # Zotu103), and *Haemophilus parainfluenzae* (ASV sequence # ZOtu122) were also identified as predictors that were less abundant in Endo+. ASV sequence # Zotu45 was initially assigned as *Malacoplasma microti*, a species previously isolated from the respiratory tracts of prairie voles (45) and an unlikely component of the CVM. Subsequent examination determined that the assigned ASV shared 100% identity with uncultured *Mycoplasma* sp. clone Mnola (46), now *Ca*. *Mycoplasma girerdii* (47), a strict endosymbiont of *Trichomonas vaginalis* (TV). Within TRAC, we observed that the detection of *Ca*. *M. girerdii* ASV reads coincided with a positive TV clinical diagnostic finding in 14 of 37 participants (37.8%), but was only detected in 7 of 183 (3.8%) of participants who tested negatively for TV, suggesting that this was the more correct assignment. Overall, these results suggest that multiple CVM features together can be predictive of susceptibility to CT ascension, but the amount of CT at the cervix is a key factor.

**TABLE 1:** ASVs predictive of ascended CT infection^*a*^

Relative abundance	Prediction approach	Species	ASV	Selection frequency	Total read counts	%ID	Genus	Confidence level		Post prob	RDP based
								Genus	Species		
Higher in Endo+	With CT	*Chlamydia_trachomatis*	ZOtu76	100	15,593	100	*Chlamydia*	1.00	0.96	0.48	False
	Shared	*Acetothermia_genera_ incertae_sedis_clone*	ZOtu233	92	312	79	*Acetothermia_genera* *_incertae_sedis*	0.01	0.00	0.29	True
		*Butyrivibrio_hungatei*	ZOtu1808	99	325	89.4	*Butyrivibrio*	0.20	0.03	0.41	True
		*Syntrophococcus_sucromutans*	ZOtu2665	99	155	92.5	*Syntrophococcus*	0.26	0.07	0.49	True
Lower in Endo+	Shared	*Actinobaculum_massiliense*	ZOtu567	99	63	100	*Actinobaculum*	0.89	0.61	0.99	False
		*Actinotignum_schaalii^b^*	ZOtu281	100	200	100	*Actinotignum*	0.96	0.77	0.99	False
		*Haemophilus_haemolyticus*	ZOtu103	93	6,697	100	*Haemophilus*	0.96	0.76	0.99	True
		*Haemophilus_parainfluenzae^c^*	ZOtu122	86	3,507	98	*Haemophilus*	0.76	0.55	0.78	False
		*Ca_Mycoplasma_girerdii* ^*d*^	ZOtu45	90	68,190	100	*Malacoplasma*	0.82	0.44	0.23	True
		*Porphyromonas_bennonis*	ZOtu373	100	132	99.2	*Porphyromonas*	1.00	1.00	0.23	True
		*Propionibacterium_lymphophilum*	ZOtu333	100	141	100	*Propionibacterium*	1.00	1.00	0.24	True
		*Sutterella_sp900762445*	ZOtu184	100	690	100	*Sutterella*	0.98	0.96	0.98	False
		*Sutterella_sp900762445*	ZOtu110	98	4,343	96	*Sutterella*	0.89	0.55	0.63	False

^*a*^ The ASVs were identified as microbial biomarkers for predicting susceptibility to ascending CT infection. Selection frequency indicates the number of times an ASV was detected with non-NA values of mean decrease in accuracy, occurring in 9 or 10 out of 10 folds, out of 100 replications. These ASVs were determined based on the selection frequency exceeding 80 (see Materials and Methods for detail). Notably, despite considering two different scenarios ([i] prediction with all ASVs including CT ASV and [ii] prediction with all ASVs but excluding CT ASV), 12 out of 13 ASVs were consistently identified across both scenarios and indicated by “Shared” in the Prediction approach column of Table 1. The relative abundance of these ASVs was determined using t statistics, indicating whether an ASV was higher in Endo+ or lower in Endo+. Total read count was calculated for all 220 women, and details of the %ID Confidence level columns were provided in Table 1. The Post prob column indicates the posterior probability of the taxonomy assignment based on vSpeciateDB, and whether the taxonomy was assigned based on RDP DB is indicated in the RDP based column.

^*b*^ ASVs were assigned to a specific species from among different species that share the same sequence in the V4 region of the 16S rRNA.

^*c*^ ZOtu122 ASV was assigned as *Haemophilus influenzae* based on vSpeciateDB with 98% identity, but the ASV sequence matches to *Haemophilus parainfluenzae* with 100% identity.

^*d*^ ZOtu45 ASV was initially assigned as *Malacoplasma microti* based on RDP Classifier DB with 89% identity (confidence level: 0.8209 at the genus level and 0.4351 at the species level), but it was later identified as *Candidatus Mycoplasma girerdii* with 100% identity.

### Cervicovaginal microbial features associated with ascending CT infection are positively correlated with cytokines in cervical secretions associated with endometrial CT infection

**Fig 4:**
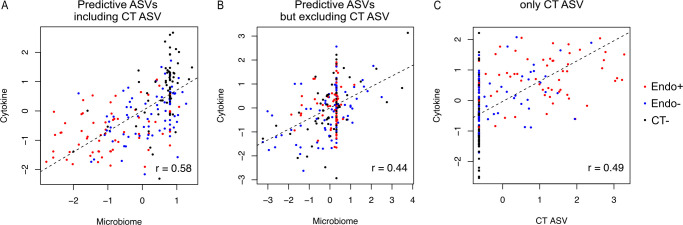
The correlations between canonical variates of microbiome biomarkers and cervical cytokines. The canonical correlations between cervical cytokines related to chlamydial ascension and microbial biomarkers identified for chlamydial ascension risk were performed under two scenarios: correlating the seven cervical cytokine levels (**A**) with 13 informative ASVs, including CT ASV, listed in Table 1, and (**B**) with 12 informative ASVs, excluding CT ASV. (**A**) The canonical correlation between the seven cervical cytokine levels and the 13 ASVs, including CT ASV, was visualized using two CCA variates, resulting in a correlation coefficient of 0.58. (**B**) The canonical correlation between the seven cervical cytokine levels and the 12 ASVs, excluding CT ASV, yielded a correlation coefficient of 0.44. (**C**) Only CT ASV abundance was correlated with the seven cytokine levels, resulting in a correlation coefficient of 0.49.

To better understand possible functional consequences of these 13 CT ascension-related microbial features on host immune responses, we explored their associations with levels of seven cytokines in TRAC cervical secretions that had been previously associated with CT ascension (48). The following cytokines, CXCL10, TNF- α, IL-17A, CXCL9, CXCL11, CCL4, and CXCL13, were positively associated with endometrial infection (unadjusted *P* < 0.05) in a univariable regression model. Using canonical correlation analysis (CCA) (49), we investigated whether there was an overall correlation between these cytokine levels and the predictive microbial features. With 13 ASVs, including CT, we observed a positive correlation between microbial abundances and cytokine levels (Fig. 4A; canonical correlation = 0.58). There was also a positive but weaker association between cytokine levels and CVM-derived ASVs when CT ASV was excluded (Fig. 4B; canonical correlation = 0.44). CT ASV abundance also correlated weakly with the seven cytokines (Fig. 4C; canonical correlation = 0.49). Together, these results indicate that host immune responses are not exclusively modulated by CT abundance. The CCA-based analyses suggest that the predictive CVM microbial features influence CT ascension directly and indirectly, through interaction with host immune responses.

## DISCUSSION

The TRAC cohort provided a unique opportunity to investigate CVM associations with chlamydial ascension from the cervix to the upper genital tract. Recent literature has revealed that cervicovaginal microbiota may modulate susceptibility to infections such as HPV, CT, NG, and MG infections (50). CVM composition has been associated with increased risk of incident CT infection (16, 22, 50, 51). In this study, we sought to determine whether CVM composition might facilitate CT ascension from the cervix to the upper genital tract by profiling 16S rRNA genes obtained from vaginal swabs collected at enrollment from the TRAC cohort. Although we were unable to identify many significant differences between CT-infected and -uninfected women, we identified 13 cervicovaginal microbial features that predicted active upper-tract infection. Chlamydial cervical burden was a key predictor of ascent because vaginal CT ASV abundance alone effectively predicted upper genital tract spread with AUC of 0.72 for sensitivity and specificity. We also observed a positive correlation between levels of seven cytokines previously associated with CT ascension and abundance of CVM-derived ASVs, indicating that local host immune responses during infection were not exclusively modulated by pathogen load.

TRAC comprised women at elevated risk for STI, and a striking feature of this cohort is the very high rate of CT infection, 67%, among enrollees, reflective of highly targeted recruitment strategies (28). This may have blunted our ability to detect significant differences in the composition of the CVM, at the level of CST, for individual genera or species that were associated with protection against CT infection. Unsurprisingly, most participants were assigned to CST III or CST IV, previously recognized as neutral or favorable for CT infection (7, 8, 11–13). Within CST III, CT− women were separable from CT+ using sPLS-DA components containing *L. crispatus*-assigned ASVs. Among CST I-assigned participants, the CST most highly associated with protection from CT infection (8, 13, 22), CVM composition did not differ sufficiently to cluster CT-infected and -uninfected participants effectively. TRAC was designed as a longitudinal study to investigate T cell responses important for protection from incident chlamydial infection, but the high rate of CT positivity at enrollment, combined with a stringent requirement that participants complete a minimum of three follow-up assessments, provided insufficient power to confirm previously identified associations of CST with infection susceptibility. Nevertheless, we were able to observe overall differences in CVM compositions between Endo− and Endo + participants, although discriminatory resolution dropped considerably when the CT ASV was excluded, emphasizing the likely importance of CT cervical abundance in driving ascension. At the level of CST, discriminatory performance was highest in CST IV, but much reduced for CST I and CST III participants, suggesting that CVM-derived factors that promote susceptibility to CT infection also contribute to its ascent. Directly relevant to our study, recent work by Hinderfield and colleagues (52) demonstrated that CST IV-associated microorganisms promote paracellular permeability of human ectocervical monolayers with coincident down-regulation of zonulin and occludin expression, leading them to hypothesize that undermining cervical epithelium integrity enhances susceptibility to TV and other STI pathogens (52). Thus, a CVM that contributes essential substrates and improves pathogen access to the cervical epithelium can increase susceptibility to chlamydial infection.

In contrast, lactic acid produced by *Lactobacillus* sp. directly kills CT (23), while D(−) lactic acid produced by some *Lactobacillus* sp. inhibits CT infection by down-regulating host epithelial cell proliferation (6). In this study, we were unable to detect associations of the most abundant *Lactobacillus* species with CT positivity but identified differences in low-abundance strains (Table S4). We questioned whether the low-abundant *Lactobacillus* ASVs that were differentially abundant by MaAsLin2 analysis were genuine *Lactobacillus* species or accidental assignments of artificial sequences to *Lactobacillus* species, given the taxonomic complexity among *Lactobacillus* species (53) and the limitations of characterizing these species through the V4 region of 16S rRNA gene sequencing (54). Although ASVs have intrinsic biological meaning independent of reference database or the specific context of a study (55), the taxonomic resolution is much lower compared to the full length of 16S rRNA sequences or metagenome sequences (56). Recent genomic studies identifying genetic elements that control functional differences between and within *Lactobacillus* species, such as the role of bile salt hydrolases that promote colonization of vertebrate-adapted niches (57), the ability to metabolize extracellular glycogen important for microbial growth in the female genital tract (58, 59), and the identification of novel urogenital tract species (60), suggest that it is premature to conclude that the differences we detected between CT-infected and -uninfected women are artifactual, and future investigations should consider implementing typing approaches with superior resolution that will enable us to determine if further investigation into the biological roles of individual *Lactobacillus* subspecies in cervical CT infection is needed.

Many of the CVM features selected by our analysis have not previously been associated with human chlamydial infection but have been investigated in the context of the respiratory or gut microbiome. Studies, *in vitro* (14, 15) and *in vivo* (16, 17), have associated indole producers, e.g., *Prevotella* sp., with CT evasion of IFNγ-mediated tryptophan depletion (18, 19). Of the *Prevotella* sp. discriminating CT infection in our analysis (61), only *P. brunnea* is an indole producer (61, 62), but it is also possible that they modulate host factors, indirectly influencing chlamydial susceptibility. *S. sucromutans*, in association with *Prevotella* sp., serves as a biomarker of gut barrier dysfunction (leaky gut) (63), where increased luminal abundance of zonulin-1, an endogenous tight-junction protein, was associated with decreased abundance of these species. It would not be surprising that conditions that increase susceptibility to chlamydial infection could also accelerate its spread to the upper genital tract.

Through “nutritional immunity,” the host may limit iron availability as a means of protection against invading pathogens (64). CT requires iron (65, 66) and may experience additional competition for this essential nutrient from CVM members. Iron restriction also impacts host cellular metabolism, leading to differential host gene expression with downstream consequences for the availability of other key substrates required for chlamydial developmental success (67). Antithetically, transcription of the *trpBA* operon essential for chlamydial scavenging of exogenous indole to tryptophan is iron-regulated (68), leading to the proposal that a low iron environment could increase risk for sequelae (68). *H. haemolyticus* is a respiratory tract commensal that limits infection with the pathogenic *H. influenzae* through competition (69) because it uses its superior ability to sequester iron to facilitate occupation of their shared niche (70, 71). The related opportunistic pathogen, *H. parainfluenzae,* expresses a hemophilin, HphA, that is structurally similar to that of *H. haemolyticus* (72). Thus, their relatively lower abundance in Endo+ participants may indicate that chlamydial need for iron predominates during natural infection and that *H. haemolyticus* and/or *H. parainfluenzae* abundance could modulate infection ascent by negatively influencing chlamydial growth.

In contrast, other predictors identified in this analysis may act as immune modulators, influencing the host’s ability to control infection, rather than through alteration of nutrient pools. For example, an ASV contributing to the prediction of ascended CT infection that was less abundant in Endo+ women identified *Actinobaculum massiliense,* a urinary tract commensal or opportunistic pathogen (73), and reportedly a rare cause of PID (74). A recent proteomic analysis identified two highly expressed subtilisin-like proteases which may cleave cytokines (75), providing potential to modulate the host inflammatory response to itself and CT. Also less abundant in Endo+ participants, *Sutterella* species have not been previously described as a significant member of CVM, but within the human gut microbiome, its reduced abundance has been identified as a predictor of enhanced susceptibility to respiratory infection (76). *Sutterella* spp. are epithelium-adherent (77), but do not contribute to gut dysbiosis or loss of gut epithelial integrity. In the context of STI, their abundance is significantly increased in the rectum of HIV-negative, STI-uninfected men, and the CD4/CD8 ratio was positively correlated with *Sutterella* in HIV-infected patients, consistent with an association with T cell-mediated adaptive responses independent of active bacterial infection (78). They are mildly proinflammatory, and it has been proposed that their association with their human host is mutualistic through their contribution to steady-state induction of Th17 cell differentiation (79, 80). We previously reported that IL-17A transcript was significantly increased in the blood of participants with high cervical CT burden compared to those with low cervical burden and uninfected participants (26), and that cervical secretion of IL-17A was associated with increased odds of endometrial infection (48). Nevertheless, we observed increased frequencies of CD4 Th17 cells in peripheral blood of CT-infected TRAC participants compared to uninfected participants, and a further increase in participants without recurring infection compared to those who were reinfected with CT within a year after enrollment (81). Together, these results suggest that CT infection elicits local and systemic Th17 responses, and that the systemic Th17 responses detected were associated with reduced reinfection. It is possible that the occupation of a niche by benign *Sutterella* within the CVM of Endo− participants protects against the expansion of CVM members that positively contribute to chlamydial burden by provision of necessary intermediates or by compromise of epithelial integrity. It is also possible that its reduced abundance in Endo+ women contributed to reduced CD4 T cell activity, ultimately delaying the resolution of chlamydial infection. Future studies that are more informative of function (e.g., transcriptomics, metabolomics) may identify common metabolites or pathways that can be associated with CT spread to the upper reproductive tract, while profiling of adaptive immune cell populations recruited to the infection site will lend further insight into mechanisms of chlamydial clearance.

We previously observed the importance of CT/NG coinfection on the risk for CT reinfection (28). We also reported altered bloodborne transcriptional signatures pointing to suppressed host adaptive responses with CT/NG coinfection in women with pelvic inflammatory disease (26). However, we did not observe TV nor MG coinfection associating with ascended CT in TRAC participants (28, 31). In this study, an ASV that we ultimately reassigned to *Ca*. *M. girerdii* (47, 82) was identified as a CVM feature predictive of ascended infection. Members of this large and diverse class of *Mollicutes,* distinguished by a small genome and lacking a cell wall, such as *Mycoplasma hominis* and *Ureaplasma* sp., have been identified as commensals and/or opportunistic genital tract pathogens, although meta-analyses have not effectively resolved the extent of their impact on female infertility or other reproductive outcomes including preterm birth (83, 84). Intriguingly, *M. hominis* and *Ca*. *M. girerdii*, alone or together, can live intracellularly or on the surface of TV, accelerating the parasite’s growth (85, 86) and modulating its proinflammatory potential. Relevant to CT infection because of the importance of cell-mediated responses for chlamydial clearance, TV harboring *M. hominis*, but not alone, synergistically upregulates IL-8, IL-1β, and TNF-α production, as well as induces production of the Th17-polarizing cytokine IL-23 by human monocytic THP-1 cells with coincubation (87). If, as we speculate, recruitment of chlamydia-specific Th1 and Th17 cells to the genital tract is essential for control, TV coinfection, in some but not all instances, might promote this outcome, explaining why *Ca*. *M. girerdii* ASV was associated with Endo– women. Confusingly, ASVs assigned to the other TV endosymbiont *M. hominis* were also detected in many TRAC participants but were not selected as predictive features. We were not able to immediately resolve this contradictory observation because rRNA from TV was not captured in this analysis since it is a protozoan, and the clinical diagnostic was not quantitative. But it highlights the potential for factors that influence pathogen success to be mediated through interactions between microbiome members, including those that cannot be detected using a 16S rRNA profiling approach.

ASVs assigned to *Butyrivibrio hungatei* and the genus *Acetothermia* were also more abundant in Endo+ participants. Neither bacterium is well documented as a member of CVM. In the context of the gut microbiome, *B. hungatei* is thought to act positively because reduced abundance of this butyrate producer has been associated with dysbiosis and disorders of the gut-brain axis such as Alzheimer’s disease (88). Butyrate is an SCFA that modulates colon health by multiple mechanisms. In low amounts, it serves as a carbon source for other commensals, while at higher doses, it can be toxic, targeting other members of normal microbiota or pathogens such as *Salmonella* sp. (89). Colonic enterocytes transport butyrate as a preferred substrate (90), promoting epithelial health and integrity. However, in the female genital tract, SCFAs are associated with the production of proinflammatory cytokines and perturbation of epithelial integrity (91).

With potential translational impact, random forest analysis revealed the importance of cervical CT abundance in the prediction of concomitant upper genital tract spread. Using the CT ASV alone is a highly informative and simple feature, capable of predicting chlamydial ascension with equivalent AUC performance to the prediction using 13 ASV CVM biomarkers. A major barrier to preventing CT disease is delayed diagnosis, because infection is often asymptomatic (92), but both clinical and subclinical upper-tract inflammation can lead to chronic oviduct damage (93). Diagnostic biomarkers of ascended infection would be particularly useful for evaluation of therapeutics and vaccines (94–96) and improve case management, since women with clinical PID receive a longer antibiotic course compared to asymptomatic women with CT (97). Abundance of the CT ASV appears to be a better predictor of ascended CT infection than bloodborne transcriptional biosignatures (98) or profiling of circulating immune cells (81) for this cohort of highly exposed women. Nevertheless, CT ASV with the CVM-derived selective features provided the best correlation with local cytokines, indicating the potential for further improved prediction with the inclusion of host immune features. We previously performed a small pilot study that indicated that whole transcriptome sequencing analysis of ribosomal RNA-depleted total RNA isolated from cervical swab samples contained pathogen-specific sequences from women with confirmed sexually transmitted bacterial pathogens (99). Simultaneously, we identified and quantified their active microbial communities, and after integration with their associated host-derived reads, we detected clustered host transcriptional profiles reflecting microbiome differences and STI. Together, these findings indicate that cervical sampling can yield sensitive and robust biomarkers for the evaluation of candidate chlamydial vaccines, such as those currently in development or advancing toward phase I (100) and phase II trials.

High-throughput sequencing of 16S rRNA enabled us to efficiently identify CVM communities in the TRAC cohort, but taxonomic resolution for microbial species or strains was frequently too low to confidently determine their taxonomy as cervicovaginal microbiome. Nonetheless, we were able to detect STI pathogenic microbes, as well as highly variable microbes such as *Lactobacillus* sp. and *Mycoplasma* sp., using ASV sequences. Copy number of the 16S rRNA gene can vary widely, ranging from 1 to 37 in bacteria and 1 to 5 in archaea (101). This variation in 16S rRNA gene copy number (GCN) limits the accuracy of quantifying 16S rRNA genes and CT 16S reads, potentially introducing biases in subsequent analyses (102). Indeed, this effect may be reflected in our variable success detecting ASVs associated with NG (four copies), CT (two copies), and MG (single copy) (103) in samples from women testing positive with highly sensitive clinical diagnostic tests. Although novel methods have been developed to correct for 16S rRNA GCN (104–107), we did not apply 16S rRNA GCN prediction tools for precise quantification of 16S rRNA genes in this study. In future work, we can adopt full-length 16S rRNA sequencing as an alternative to short reads of 16S rRNA to improve taxonomic resolution and quantification accuracy.

This study had several additional limitations. For example, the participants of the TRAC cohort were highly exposed to CT infection, which may have introduced bias in the representation of the CVM communities in general. Vaginal swab samples for microbiome sequencing were collected only at enrollment when participants were actively infected, so there were no baseline samples to investigate temporal changes in CVM features related to the development of CT ascending infection. Even if we had chosen to sample participants longitudinally, all received antibiotic treatment at enrollment, which could have affected CVM composition. Also, 16S rRNA-based sequencing provided information only on the composition of CVM profiles. This limits the ability to understand the functional roles that these microbial communities may play in modulating host immune resistance or susceptibility to CT ascension. We did not investigate other factors such as host genetic heterogeneity and the effect of innate immunity on ascended CT infection.

In future studies, the integration of other techniques, such as transcriptomics and metabolomics, with 16S rRNA-based sequencing, studies that have been initiated with the establishment of an additional cohort of CT-infected women (TRAC2) (108), is expected to facilitate the development of deeper functional insights and the identification of relevant biological pathways. Additionally, expanding the analysis with other relevant members of the CVM, including parasites such as TV and viruses, could also enhance our understanding of microbial interactions with the host and improve the prediction of upper genital tract infection spread. These multi-omics analyses may also contribute to the identification of biomarkers of protection or disease risk that could contribute to the assessment of vaccine efficacy in future clinical trials.

## MATERIALS AND METHODS

### TRAC cohort

This cohort was composed of mainly young (median age, 21 years; range, 18–35 years), single (89%), African American (66%) women at high risk for the acquisition of CT (Table S1). Inclusion criteria indicating high-risk status included >3 sexual partners in the previous 6 months, ≤14 years of age at sexual debut, history of PID, or presentation to the recruitment site with any of the following: presence of mucopurulent cervicitis on exam or sexual contact with a partner known to be infected with CT or NG or non-gonococcal non-chlamydial urethritis. Women with a current diagnosis of PID according to the Centers for Disease Control and Prevention guidelines were excluded. Additional exclusion criteria were pregnancy, uterine procedure or miscarriage in the preceding 60 days, menopause, hysterectomy, antibiotic therapy in the preceding 14 days, and allergy to study medications. A total of 246 TRAC participants were enrolled into a longitudinal study designed to investigate T cell responses important for protection from incident chlamydial infection over 12 months of follow-up between February 2011 and August 2014 and were recruited from the Allegheny County Health Department’s Sexually Transmitted Diseases Clinic, Magee-Womens Hospital (MWH) Ambulatory Care Clinic, and the Reproductive Infectious Disease Research Unit at MWH in Pittsburgh, PA. Clinical, histological, and microbiological testing was performed, and blood and endometrial biopsy samples were obtained at enrollment, after which all participants received single-dose agents to treat gonorrhoea (ceftriaxone, 250 mg intramuscularly) and chlamydia (azithromycin, 1 g orally). Participants in this cohort were assessed for cervical and endometrial infection using Aptima Combo 2 (Hologic, Marlborough, MA) NAAT with overall infection rates of 67% and 8.5% for CT and NG, respectively. MG and TV infection was determined using Aptima MG and Aptima TV diagnostics (Hologic), respectively.

### Sample collection and experimental procedures

Cervical swab samples and endometrial tissue specimens were collected at enrollment as previously detailed (28). Endometrial sampling was performed as follows: after cleaning the cervix with Betadine, a sterile endometrial sampler (Unimar Pipelle de Cornier; Cooper Surgical, Shelton, CT) was placed into the endometrial cavity, and a tissue sample was collected. The tissue was discharged into a sterile Petri dish after the sample device was removed. Tissue most proximal to the sampling portal of the cannula was fixed in 10% formalin, and adjacent tissue was placed in RNAlater solution (Thermo Fisher Scientific). Swab-absorbed material from 5 mm of tissue most distal to the portal, minimizing risk of contamination from cervicovaginal microorganisms, was used for diagnostics. DNA was extracted from reserved cervical or endometrial swab eluates using a Quick-DNA Universal Kit (Zymo Research, Irvine, CA) according to the manufacturer’s protocol. Abundance of chlamydial DNA-extracted material was determined by qPCR with SsoAdvanced SYBR mix (Bio-Rad) and primers directed against chlamydial 16S ribosomal DNA, using a CFX iCycler (Bio-Rad) (109). Each specimen was assayed in triplicate. Cervical secretions collected at enrollment were eluted for multiplex protein assays as previously detailed, and cytokines were assayed using Milliplex Magnetic Bead Assay Kits (Millipore Sigma) (48) at the Duke Regional Biocontainment Laboratory Immunology Core Unit, according to the manufacturer’s instructions, using a BioPlex 200 Luminex bead array reader (Bio-Rad).

### 16S library preparation and sequencing

DNA was extracted from cervicovaginal swabs stored at −80°C using the AllPrep DNA/RNA Mini Kit (Qiagen, Valencia, CA). Before extraction, the manufacturer’s protocol was optimized with minor modifications to achieve the best performance in terms of DNA/RNA yield and equal recovery of six different species comprising the ATCC Vaginal Microbiome Whole Cell Mix (MSA-2007). Average yields were 52.8 ng/µL yield of DNA in a final volume of 100 µL per sample. The V4 regions of the 16S rRNA gene were amplified using the Illumina 16S V4 primer set of 515F (GTGYCAGCMGCCGCGGTAA) and 806R (GGACTACNVGGGTWTCTAAT) (110, 111) designed for dual indexing as described by the Earth Microbiome Project protocol (https://earthmicrobiome.org/protocols-and-standards/16s/). Sequencing libraries were prepared according to Illumina’s 16S Metagenomic Sequencing Library Preparation Guide (https://support.illumina.com/downloads/16s_metagenomic_sequencing_library_preparation.html; part # 15044223 Rev. B), and sequenced on an Illumina NextSeq 500 Sequencer (Illumina, San Diego, CA).

### 16S rRNA-seq data processing

The 16S rRNA data analysis pipeline is outlined in Fig. S8. Raw fastq files containing paired-end read sequences per each sample were demultiplexed and preprocessed into ASVs using bioinformatics software, USEARCH v.11.0 (112) and PEAR (113). The fastq_eestats2 function in USEARCH was used to determine if trimming was needed to optimize read merging, based on expected errors for 150 bp forward and reverse reads across different length cutoffs (100, 110, 120, 130, 140, and 150 bp), which was 150 bp for our data. Merged reads were expected to be >252 bp with short overlap nucleotides (<10 bp). PEAR, known for its robustness with short overlapping regions, was employed for read merging, utilizing a minimum overlap size of eight bases (113). Following read merging, primer sequences were trimmed from both ends, and low-quality reads were filtered at a maximum expected error rate of 1.0.

Sequence denoising (error-correcting) and the generation of zero-radius OTUs (zOTUs), referred to as ASVs, were achieved using the unoise3 function in USEARCH (112). The unoise3 algorithm prioritizes read abundance over sequence differences by processing sequences based on descending order of read abundance (114). As a result, a rare sequence can be incorporated into a highly abundant centroid sequence even if its difference is relatively high and may be discarded by chance depending on the order of pooled unique sequences (112, 114). To reproduce the same set of ASV sequences in the TRAC cohort despite this limitation of randomly discarding low-abundant sequences, we added additional steps to the standard denoising and ASV generation process. We reordered unique sequences based on read frequency, read length, entropy, and confidence level of taxonomy assignment, all in decreasing order except for read length. Considering the optimal length of the V4 region of 16S rRNA DNA to be 253 bp, we calculated the distances between 253 bp and the length of the read, then arranged the reads from the smallest distance to the longest distance away from 253 bp. Using the rearranged ~995,000 unique read sequences, we consistently reproduced the same total set of 2,777 ASVs from an average of ~151,000 reads per sample. Of 2,777 ASV sequences, the Burrows-Wheeler Alignment tool (115) aligned 569 to the human reference genome (GRCh38), and these were excluded. We also removed ASV sequences shorter than 252 bp, with 1,990 ASVs retained for further analysis.

The inferred ASVs were assigned taxonomy based on two approaches: (i) using the sintax function in USEARCH with the RDP Classifier 16S trainset No. 18 raw training database (https://sourceforge.net/projects/rdp-classifier/files/RDP_Classifier_TrainingData/) and (ii) using speciateIT with vSpeciateDB (https://github.com/ravel-lab/speciateIT) (116). Although vSpeciateDB is designed for vaginal microbial species, we found that likely vSpeciateDB is still incomplete as it did not confidently assign ASV sequences to some pathogenic taxa, such as CT or MG. Thus, we primarily used species names from the vSpeciateDB when genus names are consistent with the taxonomy assigned by RDP Classifier reference (1,645 of 1,990 ASVs). However, for the remaining 345 ASVs with discrepancies in the genus assignments between these databases, we chose the genus and species name from vSpeciateDB when the posterior probability from speciateIT was greater than or equal to 0.5. Otherwise, we used the RDP-based taxonomic assignment when the posterior probability from speciateIT was less than 0.5. As a result, 208 of these 345 ASVs were ultimately assigned based on the RDP-based assignment, and the rest were updated with speciateIT/vSpeciateDB-based assignment.

For analyses based on ASV abundances of CT+ and CT− women, 783 out of 1,990 ASVs were excluded because they had fewer than 50 read counts across CT+ and CT− groups because these low-abundance ASVs represented experimental artifacts generated during sequencing. Similarly, of 1,990 ASVs, 1,043 with fewer than 50 reads across Endo+ and Endo− women were excluded from analyses based on ASV abundances of Endo+ and Endo− women.

Multiple sequence alignment of 18 low-abundance *Lactobacillus* sp. ASV sequences (Table S4) with their corresponding sequences of the most dominant *Lactobacillus* sp. ASVs and the V4 region sequences of *Lactobacillus* sp. obtained from the RDP 16S No18 reference database were performed using MUSCLE v.3.8.31 (117). The alignment was visualized using UGENE (118) to identify mismatches in the central region of the 252 bp ASV sequences (Fig. S4).

### Modification of reference sequences for V4 regions in RDP Classifier

RDP Classifier 16S trainset No18 raw training database provides both bacterial and archaeal 16S rRNA reference sequences. Among a total of 21,195 reference sequences, nearly 40% had identical V4 region sequences for multiple species ranging from 2 to 65 different species in the RDP Classifier database (Table S7). In such cases, the sintax function for taxonomic assignment in USEARCH randomly chooses a species among multiple species given by the reference annotation (119). In addition, since the RDP Classifier database is not limited to human-related microbial taxonomy annotation, assigned species may include those not typically observed in the human cervicovaginal microbiota. For example, although *L. crispatus* and *L. jensenii* are commonly found in the cervicovaginal environment, taxonomic assignment based on the V4 region of the RDP Classifier database might incorrectly identify *L. acidophilus* as the predominant *Lactobacillus* species instead (120). To enhance the reliability of species assignments, we implemented a two-step approach. From the ASV abundance table, we selected a single species among those with identical V4 reference sequences based on the highest abundant species driven from the RDP Classifier database. Selected species are indicated with stars (*) in Table 1 and Tables S3, S4, and S6. Remaining species and their corresponding V4 reference sequences were removed from the reference database. Using this reduced set of V4 region sequences as our reference database, we repeated taxonomy assignment for ASVs. Despite random, sintax algorithm-driven selection of a species from among multiple alternatives, we were able to track the species chosen from among those with identical V4 sequences.

### Statistical analyses

#### Alpha diversity

To investigate significant differences in alpha diversity among the three groups (Endo+, Endo−, and CT−), we standardized the total read counts per sample, which ranged from 34,571 to 417,336, by rarefying the reads across samples to the minimum read counts (34,571 reads) using the rarefy_even_depth function in the phyloseq R package. We assessed Shannon (richness) and Inverse Simpson (evenness) using phyloseq and microbiome R packages. Phylogenetic diversity was calculated using the picante R package. The phylogenetic tree for phylogenetic diversity was generated from the 16S rRNA V4 region sequences of swab samples by using MUSCLE (117). Statistical significance in alpha diversity between each pair of groups was determined using the Wilcoxon rank-sum test.

#### The centered log-ratio transformation

Microbiome-derived read count data obtained through amplicon sequencing is analyzed as compositional data (121) where a proportional change for any microorganism will affect relevant abundances of others, rendering ASV read abundances mutually dependent. CLR transformation is one approach that generalizes relative abundances with respect to the geometric mean of all sequences in the sample (122). CLR transformation of read counts enables meaningful comparisons of microbial abundances by mitigating compositional data biases (123). Since CLR transformation depends on logarithms, handling zero counts is challenging. Absolute zero counts in the CLR transformation were addressed by replacing them with the smallest CLR-transformed value derived from our read counts.

#### CST classification of TRAC cohort and discrimination analysis for CT infection status associated with CST

TRAC participants (*n* = 220) were categorized into CSTs according to their vaginal microbial compositions using VALENCIA (VAginaL community state typE Nearest CentroId clAssifier) (124). VALENCIA classifies samples for CSTs based on their similarity to a set of reference centroids, and the VALENCIA tool and reference centroids were sourced from GitHub (https://github.com/ravel-lab/VALENCIA). Where necessary, the taxon names from the RDP Classifier database were manually adapted to correspond with the VALENCIA taxonomy of the reference centroids provided in VALENCIA2_CST_centroids_19Aug2024.csv (https://github.com/ravel-lab/speciateIT).

sPLS-DA (42) was employed to discriminate between CT infection status across all women and to differentiate between CT infection status within CST I, CST III, and CST IV types. The sPLS-DA was executed using the splsda function in the mixOmics R package (125). For training, 10-fold cross-validation (CV) with 1,000 iterations was used, while within assigned CSTs, sPLS-DA utilized fivefold CV repeated 1,000 times. To assess the sPLS-DA model, AUC scores were computed from the training CV sets and averaged over 1,000 iterations, yielding the mean and SD. The ASVs listed in Table S3; Table S6 were extracted using the perf function in the mixOmics R package with a threshold of frequencies greater than 0.80 for each component.

#### MaAsLin2 for differential abundance analysis

To investigate if CVM of the TRAC cohort differs in abundance between CT+ and CT− women, MaAsLin2 (43) was applied to CLR-transformed ASV abundances using the Maaslin2 R package (normalization = “NONE,” transform = “NONE,” analysis_method = “LM,” correction = “BH,” min_prevalence = 0).

#### Random forest classification

Random forest classification was used to determine if CVM excluding CT is predictive of the lack of CT infection. This method was also utilized to predict chlamydial ascending infection and to identify microbial biomarkers. The random forest was performed using the randomForest R package (126), and the processes of parameter selection and biomarker identification are outlined in Fig. S9. In the parameter selection, we selected the optimal hyperparameters to improve prediction performance. For model evaluation and biomarker identification, a random forest classification model was generated and evaluated with K-fold CV, simultaneously choosing the microbial biomarkers of chlamydial ascension.

The following parameters were examined: (i) number of folds for CV (K = 5 or K = 10), (ii) level of phylogeny used to aggregate ASVs into species or genus, (iii) significance level of *t*-tests and Wilcoxon rank-sum tests used for aggregated microbial features (0.05 or 0.10), (iv) whether to use the default setting of the randomForest function or a hyperparameter grid search. Tuning parameters in the randomForest function included three options: mtry (number of trees randomly sampled as candidates at each split), nodesize (minimum size of terminal nodes), and sampsize (sizes of sample to grow). To accomplish this, we split our data set into training and test data sets using K-fold CV, ensuring that each fold included samples from both the Endo+ and Endo− groups. With training data, we performed two-sample *t*-tests and Wilcoxon rank-sum tests on ASVs to aggregate ASVs having *P*-value <0.05 and the same direction in abundances within the same genus or species between Endo+ and Endo− groups. Specifically, when ASVs have a *P*-value <0.05, ASVs demonstrating the same directional trend in abundances (i.e., either positive or negative t-statistics) within a species or genus were aggregated into a single microbiome feature. ASVs with *P*-values >0.05 but with consistent directional trends were also aggregated into a single microbiome feature. These aggregated microbiome features were tested again using *t*-tests and Wilcoxon tests to choose microbial features having *P*-values less than the significance level (0.05 or 0.10). The aggregated microbiome features were subsequently employed as features of training data and test data for random forest classification. For every combination of the four parameters, AUC values were calculated from 20 replicates of random forest, and parameters were selected based on the highest sum of the mean and median AUC values across the 20 replicates. As a result, the optimal parameters chosen for the prediction of absent CT infection included a significance level of 0.10, a 10-fold CV, aggregation of read counts at the species level for ASVs, and a hyperparameter grid search. These settings were applied twice, once including CT ASV as a microbiome feature, then excluding CT ASV. For the prediction of ascended CT infection, the same optimal parameters were used, except that read counts were aggregated at the genus level.

With optimal parameters, the process was reimplemented for model evaluation and biomarker identification with 100 iterations. Prediction accuracy was assessed by averaging the ROCs and AUCs obtained from the test sets over the 100 iterations. Optimal thresholds for specificity and sensitivity were obtained by the pROC R package (127). For biomarker identification, ASVs were ranked based on their mean decrease in accuracy, which indicates the importance of each feature in classifying samples. The higher the mean decrease in accuracy, the more important the variable is in the model. Using this information, ASVs were selected as predictors based on their selection frequency threshold derived from random forest predictions in Table 1 and Table S5. The selection frequency was calculated by counting how often an ASV was detected with non-NA values of mean decrease in accuracy, occurring in 9 or 10 out of 10 folds, across 100 replications. ASVs were determined if their selection frequencies exceeded 80.

#### CCA

CCA focuses on finding linear combinations maximizing the correlation between two sets of multiple variables. To assess the association between cervical cytokine levels and microbial features, CCA was implemented by the CCA R package (49).

## Data Availability

Raw 16S rRNA gene sequencing data supporting the conclusions of this article is publicly available at NCBI’s Short Read Archive (SRA) under BioProject accession number PRJNA1136868.

## SUPPLEMENTAL MATERIAL

Supplemental material iai.00057-25-s0001.docx Fig. S1 to S9; Tables S1 to S3, S5, and S7.

Table S4 iai.00057-25-s0002.xlsx Significant ASVs having differential abundances between CT+ and CT- women.

Table S6 iai.00057-25-s0003.xlsx The ASVs consistently chosen for the discrimination between Endo+ and Endo- women by sPLS-DA.

ASM does not own the copyrights to Supplemental Material that may be linked to, or accessed through, an article. The authors have granted ASM a non-exclusive, world-wide license to publish the Supplemental Material files. Please contact the corresponding author directly for reuse.
